# Using Temporal Correlation in Factor Analysis for Reconstructing Transcription Factor Activities

**DOI:** 10.1155/2008/172840

**Published:** 2008-04-17

**Authors:** Iosifina Pournara, Lorenz Wernisch

**Affiliations:** 1 School of Crystallography, Birkbeck College, University of London, Malet Street WC1E 7HX, UK

## Abstract

Two-level gene regulatory networks consist of the transcription factors (TFs) in the top level and their regulated genes in the second level. The expression profiles of the regulated genes are the observed high-throughput data given by experiments such as microarrays. The activity profiles of the TFs are treated as hidden variables as well as the connectivity matrix that indicates the regulatory relationships of TFs with their regulated genes. Factor analysis (FA) as well as other methods, such as the network component algorithm, has been suggested for reconstructing gene regulatory networks and also for predicting TF activities. They have been applied to *E. coli* and *yeast* data with the assumption that these datasets consist of identical and independently distributed samples. Thus, the main drawback of these algorithms is that they ignore any time correlation existing within the TF profiles. In this paper, we extend previously studied FA algorithms to include time correlation within the transcription factors. At the same time, we consider connectivity matrices that are sparse in order to capture the existing sparsity present in gene regulatory networks. The TFs activity profiles obtained by this approach are significantly smoother than profiles from previous FA algorithms. The periodicities in profiles from *yeast* expression data become prominent in our reconstruction. Moreover, the strength of the correlation between time points is estimated and can be used to assess the suitability of the experimental time interval.

## 1. Introduction

Genes are transcribed into mRNAs which in turn are translated into proteins. Some of these proteins activate or inhibit, as transcription factors (TFs), the transcription of a number of other genes creating a complex *gene regulatory network*. The number of transcription factors is believed to be much smaller than the number of regulated genes. Moreover, most genes are known to be regulated only by a very restricted number of transcription factors. This induces a sparse connectivity matrix for the representation of the connections between the TFs and the regulated genes. Microarray experiments measure the expression level of thousands of genes simultaneously. Unfortunately, a similar method that would allow us to measure simultaneously the abundance or activities of a larger number of proteins that act as TFs is not yet available. Some progress has been made with measurements of protein abundance by flow cytometry [1] following a dozen or so proteins of interest which need to be identified in advance. Still, such experiments are less available than gene expression experiments and cannot compete in terms of the number of tracked genes. ChIP-on-chip experiments, on the other hand, provide only static binding information about transcription factors. Thus, current approaches that use microarray experiments make a strong assumption: the protein levels are proportional to the mRNA levels. This assumption is not necessarily true due to the complexity of transcription, translation, and posttranslation modification. In more recent studies, two-level networks have been studied with hidden profiles of the transcription factors at the top level and the observed expression levels of the regulated genes at the lower level. Some of these studies [2–4] are concerned with factor analysis algorithms.

Factor analysis (FA) is often used as a dimensionality reduction approach assuming that the large number of observed variables becomes uncorrelated given a much smaller number of hidden variables called *factors*. Some of the advantages of FA over principle component analysis are the incorporation of independent additive measurement errors on the observed variables, the identification of an underlying structure, and the assignment of the factors as defined entities (in our case transcription factors). Finally, in contrast to independent component analysis, the factors are not assumed to be statistically independent.

In a recent paper, [4], we examined the suitability of five FA algorithms for reconstructing both gene regulatory networks and TF activity profiles. We showed that FA faithfully reconstructs TF activity profiles as other more widely known reconstruction approaches do such as network component analysis (NCA) [5] (see also [6,7]) and a piecewise least-square (plsgenomics) algorithm [8]. The advantage of FA analysis over these algorithms is the ability to also reconstruct the connectivity matrix. NCA and plsgenomics rely heavily on the availability of connectivity information. Nonzero positions in the connectivity matrix, which describes the connections between the factors and the genes, need to be specified in advance. The algorithms then estimate the values at these positions which actually might turn out to be zero as well. This is a strong limitation since often only little information about genes regulated by specific TFs is available. A further advantage of the Bayesian FA models is that any information from literature surveys, ChIP-on-chip experiments, or sequence analyses about the underlying structure can be easily incorporated through priors.

A serious concern regarding the currently applied FA algorithms as well as NCA and plsgenomics algorithms is the lack of incorporation of any time information provided by the experiments. Actually, most available microarray data such as the *E. coli*, yeast, and *Arabidopsis* data are obtained from time series experiments. Unfortunately, the present time correlation within the TFs is ignored in the above algorithms. Time information can act as a smoothing approach on the TF profiles and thus can improve the reconstruction process. As in our previous paper, here we are still concerned with sparse connectivity matrices, but we also aim to include time correlation within the factors. For this purpose, we extend the algorithm by Fokoué and Titterington. [9], which performed well and was computationally efficient in our comparison [4], to handle time correlation information. However, the extensions we suggest can be easily applied to alternative FA algorithms analysed in [4]. If we allowed a general form for the correlation matrix between the factors, we would run into the problem of estimating a large number of unknown parameters given only a small number of data points. We investigated a number of possible correlation structures and present one that performs well on gene regulatory networks in this paper.

Other algorithms such as the linear dynamic systems or Kalman filter models have also been suggested for estimation of the parameters of a time series model with hidden states. Ghahramani and Hinton [10] presented an EM algorithm for the estimation of the parameters of linear dynamical systems. This is an extension of the factor analysis algorithm [11] that was evaluated in our previous paper and performed less well than some alternative FA algorithms, in particular a Bayesian version. A Bayesian version of an FA algorithm allows one to use sparsity priors on the connectivity matrix and also to integrate prior information regarding the system under study. More recently, Beal et al. [12] presented a state space model for the reconstruction of transcriptional networks from gene expression time series data. The focus of their algorithm is to reconstruct a complete regulatory interaction network and not only the connectivity between TFs and genes. Thus, the hidden states do not represent TFs but any hidden variables that can not be directly measured by gene expression experiments such as missing genes, protein activity profiles, and protein degradation.

Barenco et al. [13] reconstruct the transcription factor activity of p53 from time series expression profiles of known target genes and a differential equation model of gene induction. Based on the same data set and a similar induction model, Sanguinetti et al. [14] suggest the usage of Gaussian processes to estimate the activity profile of the p53 transcription factor. In both cases, only one profile is reconstructed, albeit in great detail, and with an assumed knowledge of the dependent genes.

In this paper, we show how to incorporate time information in the factor analysis approach. Factor analysis is attractive, since it is on of the most straightforward ways to link hidden transcription factor activities to observed outputs without knowledge of the connectivity. However, time series information is ignored in all the methods discussed in our previous paper. Here, we explore an extension to factor analysis that integrates time series correlation. Since some data might show very little correlation or none at all, we estimate the posterior distribution of the strength of correlation of TF activities from one time point to the next. This information is useful in several respects as we show for gene expression data for *E. coli* from [6] and for yeast from Spellman et al. [15]. Based on these datasets, we highlight some important points: (a) the correlation parameter within the factors reveals whether the time step during experimental sampling is large or small in relation to gene regulatory processes, and what the effect of this choice has on the reconstruction process; (b) our analysis also indicates that data obtained under different experimental conditions can show quite different dynamics as reflected in the correlation, and caution is required when combining such data sets for joint inference of regulatory relationships.

## 2. Approach

In this section, we describe the general factor analysis model and how time correlation information enters the model. We discuss how the model incorporates the sparsity of the connectivity matrix and discuss identifiability problems associated with factor analysis.

We denote an instance of a random vector variable with  dimensions by , with dimensions indexed by subscripts. Instances are indexed by superscripts, , . Similarly,  is a vector of  hidden variables, also called *factors*. We assume that the number  of factors is smaller than or equal to the number  of observed variables. In a factor analysis model, the observed variables are a linear combination of the factors plus a mean and an error term:(1)

where , , , and  with  an  dimensional row vector of ones.  is called the *loadings matrix* or *connectivity matrix* with each of its entries indicating the relationship between a TF and a gene, for example,  means that there is no interaction between gene  and TF . For the rest of the paper, we assume , that is, data are centralised prior to the analysis.

FA models assume that the error terms  are independently and normally distributed with mean zero and covariance matrix , , where . Thus, the probability distribution of  has density:(2)

where tr is the trace, the sum of the diagonal elements. In Section 5, we discuss in detail the prior and posterior probabilities of the parameters , and , as well as algorithms for their estimation.

### 2.1. Time Correlation within the Factors 

The key difference between this and previously published work [2–4] is in the treatment of the prior and posterior distributions of the factors. Here, we aim to include time correlation information within the factors which is essential for time series experiments. The factors are assumed to be normally distributed with mean zero and covariance matrix . However, the covariance matrix  is now decomposed into two separable matrices  and  as their Kronecker product:(3)

where the  matrix ( dimensions) is known as the *between* factors or *spatial* covariance matrix, which captures the correlation between the factors. We assign to  the identity matrix to avoid scaling problems (see Section 2.3 below). The  matrix ( dimensions) is known as the *within* factors or *temporal* covariance matrix. We assign 1 to the diagonal entries of this matrix so that it can be treated as a correlation matrix. The  matrix captures the time correlation of each factor across the time points. We assign a first-order Markov structure to , thus an extra parameter  that indicates the correlation between neighbouring time points must be estimated. The prior and posterior distributions of matrix  as well as of the other parameters are discussed in Section 5.

### 2.2. Sparsity on the Connectivity Matrix 

Most genes are regulated by a small number of transcription factors. This implies that the connectivity matrix  is sparse. To incorporate this sparsity in the factor analysis model, we assign independent Gaussian priors to each element  of  with mean zero and variance . A Gamma prior is assigned to each  leading to a Student *t*-distribution for each row of . Such distribution assigns most probability mass to the origin and along the spines, where one of the coefficients  is zero, that is, this prior favors connectivity structures in which genes are regulated by a small number of TFs.

### 2.3. Identifiability Problems

There is an identifiability problem associated with (1). For a  dimensional orthogonal matrix  (i.e., ), we have  with . Hence, it is not possible to distinguish between  and all its possible orthogonal transformations  based on knowledge of the product  alone. However, with the objective of constructing a sparse connectivity matrix the number of possible orthogonal transformations is highly restricted.

Orthogonal transformations also include permutations of the factors which, in the case of regulatory networks, limit our ability to assign factors to known TFs. Sabatti and James. [3] constrain the factors to known TFs by assigning a priori loadings matrix with a large number of zeros based on the Vocabulon algorithm [16]. Here, we aim to see if the FA algorithm is also able to reconstruct the activity profiles without any prior information. To assess how well profiles are reconstructed, we match inferred factors to TFs using profiles from the NCA algorithm, which requires prior knowledge of the regulatory relationships. For the matching of profiles, we use the Hungarian algorithm [17] described in Section 5.

In the FA algorithm, we utilise a Gibbs sampling algorithm in order to estimate the unknown parameters. In each iteration of the algorithm, it is possible that factors change sign or change position in the factors matrix. This is a widely known problem in FA. Here, we suggest the use of the Hungarian matching algorithm to identify factors that have changed sign or position in the factors matrix.

## 3. Results and Discussion

We analysed two-gene expression datasets, one from *E. coli* and one from yeast, both from time series experiments.

### 3.1. *E. coli* Dataset

We have previously analysed the *E. coli* dataset using FA algorithms [4]. The reconstructed profiles were rugged since the temporal information was ignored. Here, we reanalyse the data (consisting of 16 TFs, and 100 gene expression levels over 24 time points) using the new algorithm, and we compare the results with the previous ones in [4]. We first approach the problem by supplying the connectivity matrix used in [6]. This connectivity matrix was constructed based on RegulonDB and the available literature, and we will refer to it as the Kao connectivity matrix. Note that the NCA algorithm (or GNCA, a variation of it) requires such information in order to reconstruct the factor profiles and to estimate the nonzero values of the factor loadings matrix. Each algorithm was run 10 times. For the GNCA algorithm, we consider the run with the least mean-squared error, while for the FA algorithms we consider the average of these runs.

**Figure 1:**
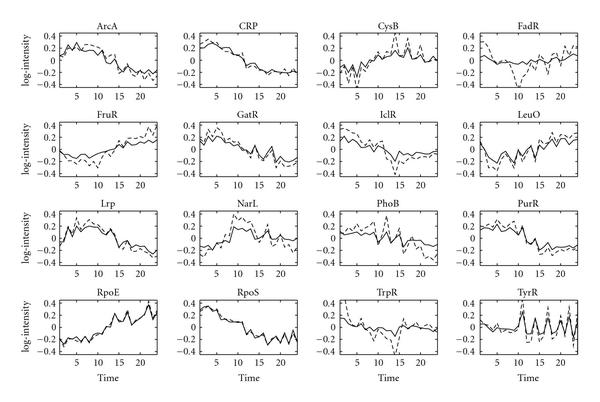
**Reconstructed TF profiles for the *E. coli* dataset with zero entries fixed in the factor loadings matrix**. GNCA profiles are indicated by the dashed lines, and FA profiles without time series correlation are shown with solid lines. Each subfigure corresponds to one of the 16 TFs. The name of the TF that is reconstructed is indicated on top of each subfigure. The *x*-axis indicates the experiments (time points), and the *y*-axis indicates the activity levels of a TF.

Figure 1 shows the TF profiles reconstructed by GNCA and FA algorithms discussed in [4]. This figure is slightly different from the one presented in [4] due to the different prior parameters that are used for the noise covariance matrix (see Section 5.6). The reconstructed profiles are rugged for both algorithms. Note that the GNCA profiles in [5] are smooth only due to some further processing steps.

**Figure 2:**
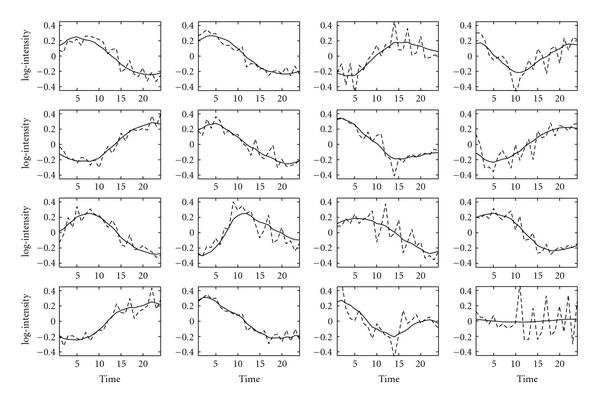
**Reconstructed TF profiles for the *E. coli* dataset with zero entries fixed in the factor loadings matrix**. GNCA profiles are indicated by the dashed lines, and time series FA profiles are shown with solid lines. Each subfigure corresponds to one of the 16 TFs as described in Figure 1.

Figure 2 shows the profiles obtained by the suggested time series FA algorithm. Overall, the profiles follow the same trends shown in Figure 1. However, the reconstructed profiles are now smooth due to the incorporation of temporal correlation. The posterior mean of the correlation parameter  is 0.85 (see Section 5). This value was obtained with a broad prior.

We also analysed the influence of the prior on the reconstruction process by providing prior loadings matrices that are denser than the Kao connectivity matrix, that is, we run the FA algorithm with decreasing numbers of given zero position in the prior loadings matrix. We tested priors where 75, 50, 25, or 5 entries per TF were fixed to zero. These positions were chosen randomly out of the approximately 75 zero positions that are present for each TF in the Kao connectivity matrix. We found that even with 50 zero entries out of the 75 entries that are present in Kao connectivity matrix the FA algorithms can reconstruct the same TF activity profiles equally well without having an identifiability problem regarding the labelling of the factors. However, as the number of constraints to zero in the prior loadings matrix is reduced further, assigning factors to TFs becomes more difficult, although the reconstruction of the TF profiles is still acceptable. This result is interesting from a biological perspective since it shows the importance of integrating prior information regarding the system under consideration even if the available information is limited. Negative experimental results showing that there is no connection between a TF and a gene are even more informative than positive experiments for this kind of analysis since they restrict the structure of the connectivity matrix very effectively.

**Figure 3:**
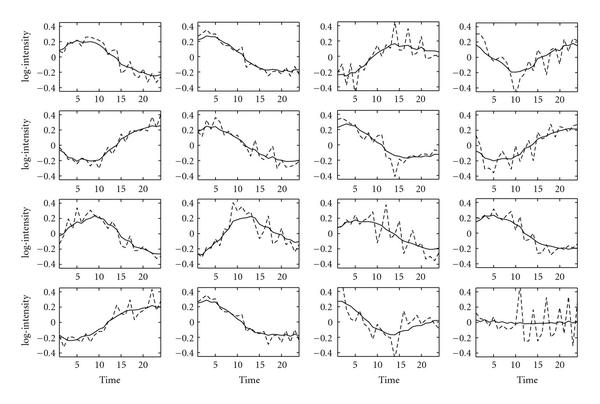
**Reconstructed TF profiles for the *E. coli* dataset without fixing the zero entries in the factor loadings matrix**. GNCA profiles are indicated by the dashed lines, and time series FA profiles are shown with solid lines. Each subfigure corresponds to one of the 16 TFs as described in Figure 1.

Finally, we analysed the *E. coli* expression data without any prior information. The reconstructed profiles strongly resemble the profiles when prior information is provided, as shown in Figure 3. The profiles are still smooth and the estimated  value is .86. Since it is unknown which factor corresponds to which TF, we matched the profiles reconstructed by the time series FA algorithm with the profiles obtained by the GNCA algorithm using the Hungarian algorithm [17].

**Figure 4:**
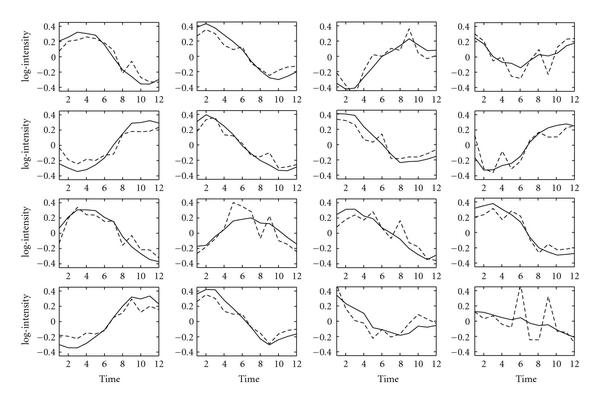
**Reconstructed TF profiles for the *E. coli* dataset with zero entries fixed in the factor loadings matrix**. The size of the original dataset is halved by removing every other sample. GNCA profiles are indicated by the dashed lines, and time series FA profiles are shown with solid lines. Each subfigure corresponds to one of the 16 TFs as described in Figure 1.

Given the *E. coli* dataset, we performed another test to assess the reconstruction process based on a sample with larger time intervals: we removed every other time point starting from the second. The new dataset consists of 12 time points instead of 24. Figure 4 shows the profiles as reconstructed by the time series FA algorithm with solid lines. The dotted lines are the benchmark profiles reconstructed by the GNCA algorithm (Figure 1), where we have now removed the missing time points. As can be seen the algorithm still reconstructs the TF profiles quite faithfully. The profiles are slightly less smooth than those in Figure 2, the effect of sampling with a larger time interval. The larger interval also causes a reduction in the estimated correlation coefficient which is now estimated as 0.71 which is consistent with the correlation from the full time series, since . The faithful reconstruction of the TF profiles is an encouraging result considering that it is often difficult to decide on a time interval with which to sample experimental data.

The above correlation and reconstruction result for the subsample suggests that the experimental sampling frequency could have been reduced to about half the time points without too much loss of accuracy. Of course, if the correlation coefficient dropped much further that would indicate that a shorter time interval is required for an accurate reconstruction of the factor profiles.

**Figure 5:**
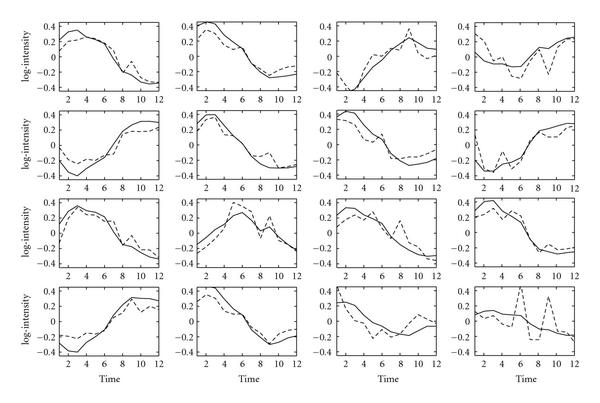
**Reconstructed TF profiles for the *E. coli* dataset without fixing the zero entries in the factor loadings matrix**. The size of the original dataset was halved by removing every other sample. GNCA profiles are indicated by the dashed lines, and time series FA profiles are shown with solid lines. Each subfigure corresponds to one of the 16 TFs as described in Figure 1.

Finally, a reconstruction with half the time points was also performed without any prior information regarding the underlying gene regulatory network. Figure 5 shows the reconstructed TF profiles after they have been matched to GNCA profiles using the Hungarian algorithm. Again, the profiles are very close to the ones reconstructed with prior information (Figure 4) indicating that prior information is not necessary in order to reconstruct the profiles. Of course, it is required if one wants to match factors to TFs. The value of the correlation coefficient was again estimated at around 0.71.

### 3.2. Yeast Dataset

We also apply the proposed algorithm to gene expression data from yeast [15], a dataset which has been used to evaluate the NCA algorithm by Kao et al. [6]. They reduced the initial dataset of 2200 genes and 113 TFs to 441 and 33, respectively. This reduction in the size is required due to some network structural restrictions implied by the NCA algorithm. For comparison reasons, we also used this reduced dataset. Spellman et al. [15] used different culture synchronisation techniques: elutriation, -factor arrest, and cdc15 temperature sensitive mutant arrest. Thus, there are three datasets consisting of 14, 17, and, 24 time points, with time intervals of 30, 7, and 20 minutes, respectively.

**Figure 6:**
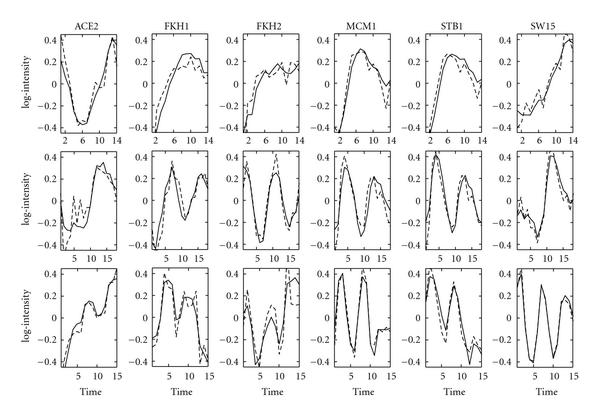
**Reconstructed TF profiles for the spellman yeast dataset with zero entries fixed in the factor loadings matrix**. GNCA profiles are indicated by the dashed lines, and time series FA profiles are shown with solid lines. The first row corresponds to the elutriation time series, the second row to the -factor arrest time series, and the third row to the cdc15 temperature sensitive mutant arrest time series. Each column corresponds to the reconstructed profiles of a given TF under three different experimental conditions. The name of the TF is shown on the top of each column. Plotted are 6 representative TFs out of 33. The *x*-axis indicates the experiments (time points), and the *y*-axis indicates the activity levels of a TF.

We analyse each time series dataset separately given the connectivity matrix also used in [6]. Figure 6 shows the reconstructed profiles of five well-known TFs. Each row corresponds to the three different synchronisation methods. Little periodic behaviour is shown during the elutriation experiment. However, a large number of profiles show periodic profiles in the other two time series experiments. For example, STB1 and MCM1 have been shown to play a crucial role in cell cycle regulation, and their profiles show a strong periodicity.

The estimated yeast TF profiles are not as smooth as the *E. coli* profiles from the previous section. However, they are still smoother than the profiles estimated by the GNCA algorithm or the static FA algorithms. A possible explanation could be the—in comparison to *E. coli*—smaller correlation coefficients that are estimated for each time series: 0.68 for the elutriation, 0.56 for the factor arrest, and 0.45 for the cdc15 mutant arrest time series.

Figure 6 shows that different synchronisation methods result in different TF profiles and different estimations for the correlation coefficients particularly after accounting for differences in the time intervals. The comparatively large differences in the correlation coefficients hint at differences in the underlying dynamics. It seems, therefore, not advisable to combine datasets obtained under such different conditions for time series analyses based on such dynamics.

**Figure 7:**
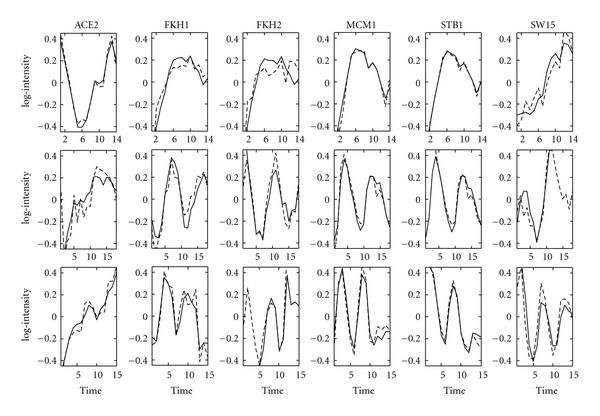
**Reconstructed TF profiles for the spellman yeast dataset without giving the positions of the zero entries in the factor loadings matrix**. GNCA profiles are indicated by the dashed lines, and time series FA profiles are shown with solid lines. The first row corresponds the elutriation time series, the second row to the -factor arrest time series and, the third row to the cdc15 temperature sensitive mutant arrest time series. Each column corresponds to the reconstructed profiles of a given TF under three different experimental conditions. The name of the TF is shown on the top of each column. Plotted are 6 representative TFs out of 33. The *x*-axis indicates the experiments (time points), and the *y*-axis indicates the activity levels of a TF.

For completeness, Figure 7 shows the reconstructed profiles of the time series FA algorithm without any prior connectivity information. Again, the TF profiles are reconstructed well. Compared to GNCA, these profiles seem smoother but they are slightly rougher than the ones in Figure 6. This is due to the greater number of parameters that need to be estimated.

Finally, we measured the correlation of the constructed TF profiles with their corresponding expression profiles without finding any significant match. This result is consistent with that of Boulesteix and Strimmer. [8].

## 4. Conclusion

We presented a factor analysis algorithm that incorporates temporal correlation within each factor vector and also learns sparse factor loadings matrices. It is quite plausible that the underlying regulatory networks are quite sparse, and reconstruction algorithms based on this principle have received increasing interest recently. There is also increasing interest in time series experiments, since they are able to capture the dynamics of biological processes. The algorithm presented here utilises both sparsity and time correlation within a Bayesian framework. We estimated the distribution of time correlation coefficients for gene expression data from *E. coli* and yeast. It seems that a fair amount of correlation is present in the data, as might have been expected. Consequently, the ragged profiles resulting from a simple factor analysis without time constraints seem to be artifacts; it is simply unlikely that activity levels fluctuate so wildly as seen for some genes. The profiles reconstructed incorporating the estimated correlation certainly look smoother, and it is likely that they are closer to the true activity profiles than the ragged reconstructions by simple factor analysis methods. For the *E. coli* dataset, we demonstrated the effect of the time interval on time correlation and reconstructed profiles. Finally, we showed that the reconstruction process is reliable even with little or no information regarding the connectivity matrix. However, some form of prior knowledge is necessary to obtain smoother profiles or to match unknown factors to known TFs.

One drawback of the GNCA and FA algorithms is that they only model linear relationships between the TFs and the regulated gene. However, as shown in the PhD thesis by Pournara [18] the assumption of linearity is not a severe one given the small amount of data and the significant amounts of noise present in microarray data. Moreover, the linearity assumption is less of a problem after mRNA abundance levels or ratios are transformed logarithmically. As theoretical considerations (see [19]) show, gene regulation by several factors might be considered additive on the logarithmic scale, unless there is severe interaction between the factors. Some discussion of this issue is also provided in [6]. Nevertheless, we are currently exploring approaches to nonlinear modelling using Gaussian processes and investigating ways to include time delays in the FA algorithms that can cause shifts to the profiles of the regulated genes. For the data used here, preliminary results using a Gaussian processes algorithm show that all factor to gene interactions can be effectively described by linear relationships.

## 5. Methods

### 5.1. Factors 

Here, we extend the methods of [4] in order to include time correlation within the factors (see [20] for a similar approach). The factors are assumed to be normally distributed with mean zero and covariance matrix , where  is the *spatial* covariance matrix and  is the *temporal* covariance matrix. The  matrix captures the time correlation of each factor across the time points. The same temporal matrix is assigned to each factor. Given the matrix identities(4)

The prior distribution of  is given by(5)

The posterior probability of the factors is now derived as(6)

where(7)

#### 5.1.1. Spatial Covariance Matrix 

The convenient conjugate prior, inverted Wishart distribution, is chosen for the spatial covariance matrix of the factors  such as the posterior distribution is also an inverted Wishart distribution given by(8)

where  is the prior hyperparameter of the Wishart distribution. As mentioned above, in this paper, we fix  to avoid some identifiability problems.

#### 5.1.2. Temporal Covariance Matrix 

General Case 

The treatment of the temporal correlation matrix  is more complicated. If we assume that the temporal matrix is completely unknown, a conjugate prior distribution is the inverted Wishart distribution with prior hyperparameter .

Thus, the posterior distribution is also an inverted Wishart distribution given by(9)

This approach greatly increases the number of parameters to be estimated to . This large number of extra parameters causes problems (singular matrix) to the Gibbs sampler, and thus we have to considerably reduce this number of unknown parameters.

First-Order Markov Structure 

If we assume that the correlation matrix  has a known structure, we can significantly reduce the number of estimated parameters. We have examined a series of different structures included the single-parameter structure and concluded that the first-order Markov structure gives the best results, and thus we will only discuss this latter case.

According to a first-order Markov structure, the matrix  is given by(10)

where . We assign the beta distribution as the prior distribution of :(11)

where . For  and , the beta distribution equals the uniform distribution. For large values of ,  approaches 1, while for large values of  approaches −1.

Given the determinant , and the inverse of (12)

where :(13)

The posterior distribution of  takes the following form:(14)

where , , , and . Note that the prior distribution of  is wrongly assigned in [[20], page 285]. Moreover, the posterior distribution is slightly different due to the difference in the treatment of the noise covariance matrix. Finally, the loadings matrix is also treated differently here (see below).

### 5.2. Factor Loadings Matrix 

Independent Gaussian priors are assigned to each element  of , . To each , a Gamma prior is assigned, . The posterior probability of each row  of  is given by(15)

where , , , and  is a row vector that corresponds to the th row of .

The posterior distribution of  is also a Gamma distribution given by(16)

### 5.3. Noise Covariance Matrix 

A convenient Gamma prior with shape parameter  and scale parameter  is assigned to the inverse of the noise covariance matrix  so that its posterior distribution is also a Gamma distribution given by (17), where :(17)

### 5.4. Gibbs Sampler

A Gibbs sampler algorithm (Algorithm 1) is used to estimate the unknown parameters. We first initialise the loadings and noise covariance matrices. Then, we iterate through steps 1 and 2 sampling from the posterior conditional distributions of the parameters. In the analysed datasets, convergence was reached within the first 100 samples. However, we discarded the first 3000 samples in order to ensure convergence and collect another 500 samples for the analysis.

**Algorithm 1:** Gibbs sampler algorithm for factor analysis.

*Step 1.* Sample  from (6),

sample  from (14) using a rejection sampling algorithm.

*Step 2.* Sample  and  from (15) and (17), respectively.

### 5.5. Hungarian Algorithm

The sign of the factors can change during the Gibbs sampling algorithm. Moreover, two factors can alternate position in the factors matrix. These are two widely known problems with factor analysis. In order to overcome these problems, we suggest the use of the *Hungarian algorithm* which solves assignment problems in polynomial time. A cost matrix of dimensions  is constructed given the inferred factor matrices at rounds  and  of the Gibbs sampler. The  element of the cost matrix, is the mean-square error between the th and th profiles of the factor matrix at time  and , respectively. This cost matrix is used to solve the assignment problem by minimising the sum of the costs. For the details of the algorithm, the reader is referred to the seminal paper by Kuhn [17]. We also used this algorithm to assign inferred factor profiles to known TF profiles, that is, reconstructed by the GNCA algorithm.

### 5.6. Prior Parameters

The choice of prior hyperparameters is very important in a Bayesian framework. We choose uninformative priors for most of the parameters. The estimation of the  parameter is fairly insensitive to the choice of the hyperparameters. Thus, we used a uniform prior distribution for the *E. coli* dataset and a prior of  and  for the yeast data. In the time series FA algorithm, the choice of the hyperparameter for the noise covariance matrix is important. While a very flat prior is a good choice in the static FA, in the time series FA analysis a prior distribution away from zero gives better results. However, we note that as long as the distribution is away from zero, the algorithm is robust with respect to the exact width of this distribution. In our experiments, the shape and scale hyperparameters were set to 10. These parameter values give a distribution away from zero and one that does not include unrealistically large values. For the  parameter, we choose shape and scale parameters 10 and 0.1, respectively, in order to favour sparse connectivity matrices. To indicate that our reconstructed profiles are not a result of the choice of the hyperparameters, Figure 1 shows the reconstructed profiles for the static FA algorithm using exactly the same hyperparameters that are used in the time series FA algorithm.
